# Failure Criteria and Constitutive Relationship of Lightweight Aggregate Concrete under Triaxial Compression

**DOI:** 10.3390/ma15020507

**Published:** 2022-01-10

**Authors:** Peihuan Ye, Yuliang Chen, Zongping Chen, Jinjun Xu, Huiqin Wu

**Affiliations:** 1 College of Civil and Architecture Engineering, Guangxi University of Science and Technology, Liuzhou 545006, China; yepeihuan@yeah.net (P.Y.); zpchen@gxu.edu.cn (Z.C.); whq6329@163.com (H.W.); 2 Key Laboratory of Disaster Prevention and Structure Safety of the Ministry of Education, Guangxi University, Nanning 530004, China; 3 School of Civil Engineering and Transportation, South China University of Technology, Guangzhou 510641, China; 4 College of Civil Engineering, Nanjing Tech University, Nanjing 211816, China; jjxu_concrete@njtech.edu.cn

**Keywords:** lightweight aggregate concrete, triaxial behavior, failure mechanism, stress-strain constitutive relationships, fly ash ceramsite

## Abstract

This paper investigates the compression behavior and failure criteria of lightweight aggregate concrete (LAC) under triaxial loading. A total of 156 specimens were tested for three parameters: concrete strength, lateral confining pressure and aggregate immersion time, and their effects on the failure mode of LAC and the triaxial stress-strain relationship of LAC is studied. The research indicated that, as the lateral constraint of the specimen increases, the failure patterns change from vertical splitting failure to oblique shearing failure and then to indistinct traces of damage. The stress-strain curve of LAC specimens has an obvious stress plateau, and the curve no longer appears downward when the confining pressure exceeds 12 MPa. According to the experimental phenomenon and test data, the failure criterion was examined on the Mohr–Coulomb theory, octahedral shear stress theory and Rendulic plane stress theory, which well reflects the behavior of LAC under triaxial compression. For the convenience of analysis and application, the stress-strain constitutive models of LAC under triaxial compression are recommended, and these models correlate well with the test results.

## 1. Introduction

Sustainable construction has been an important concept to reduce the carbon footprint and save natural resources in civil engineering. Fly ash is a byproduct of coal combustion in electric power plants and is considered an environmental pollutant [1,2,3,4]. According to statistics, approximately 600–800 million tons of coal fly ash is produced globally on an annual basis, while less than 30% of this is reused [5,6,7]. At present, the treatment of fly ash is one of the main difficulties in environmental protection, as it requires the occupation of large areas of land. The heavy metal elements (i.e., arsenic (As), lead (Pb), zinc (Zn), nickel (Ni), copper (Cu), manganese (Mn), cadmium (Cd), chromium (Cr) and selenium (Se)) [8,9,10,11] and polycyclic aromatic hydrocarbons contained in fly ash will pollute the surrounding soil, surface water and groundwater resources. Therefore, attempts to increase the recovery and utilization rate of fly ash are of great significance. One of the effective measures to use fly ash is to make ceramsite aggregate and then replace the natural aggregate to produce a lightweight aggregate concrete (LAC). Recycling and reusing fly ash activate the so-called “misplaced resource” in terms of manufacturing the fly ash ceramsite. LAC made of fly ash ceramsite has the characteristics of low weight, high heat preservation and insulation, as well as excellent seismic performance; therefore, it has good application prospects [12,13,14,15].

Recently, some scholars have explored the properties of LAC as an energy-saving and sustainable construction material. Studies showed that LAC also has some other interesting properties. First, despite its many advantages, its higher brittleness and lower mechanical properties compared to normal-weight concrete at the same compressive strength [16,17,18] prevent it from achieving wide application in the construction industry. Second, the water absorption rate of LAC is large, and the cement slurry will easily shrink and crack. Nonetheless, it can allow internal curing due to the water absorption and desorption capability of ceramsite. Therefore, the early-age autogenous shrinkage of LAC decreases with the increased pre-wetting degree of ceramsite for the same net water-to-cement ratio or total water-to-cement ratio [19]. The maximum residual stress of concrete mixture decreases with the increasing proportion of pre-wetted lightweight aggregates [20]. Considering the above adverse characteristics of LAC, it is generally improved by the addition of fibers or geopolymers. In general, the incorporation of fiber can improve the mechanical properties of LAC, significantly improve its toughness, ductility and energy absorption performance, and reduce its workability, especially when steel fiber is used [16,21]. Lightweight concrete is significantly affected by the water:binder ratio and the performance of the lightweight aggregate. The addition of fiber has little effect on the compressive strength, but significantly increases the splitting tensile strength and bending strength [22]. In addition, the use of styrene–butadiene rubber in the recently modified lightweight aggregate concrete leads to its lower water absorption and the significant improvement of its resistance to chemical attack and corrosion [23].

Due to the high brittleness and low strength of LAC, steel tubes, GFRP and spiral hooping are often used to restrain it in engineering to improve the poor mechanical properties and achieve the purpose of reducing the self-weight of the structure; however, this puts LAC in a complex stress state under multi-axial stress. The mechanical properties of LAC under triaxial stress were studied for engineering applications. Yu et al. [24] and Ren et al. [25] proved that LAC under triaxial compression showed better post-peak toughness and energy absorption capacity and found that the effect of lateral stress on LAC was significantly stronger than that of ordinary concrete. Yang et al. [26] found that lightweight aggregate concrete shows the obvious platform fluxplastic phenomenon under triaxial compression, and the strength analysis results with octahedral intermediate principal stress were in good agreement. Wang et al. [27] discussed the mechanical behavior and failure criteria of gangue-based haydite concrete under triaxial loading. Under compression, when *σ*_1_/*σ*_3_ ≥ 0.3 and *σ*_2_/*σ*_3_ ≥ 0.5, most of the ceramsite in the sample was crushed. According to the test data, a four-parameter triaxial failure criterion was established. Fu, Z.Q. et al. [28,29] studied the strength characteristics of LAC constrained by steel tubes, and the results showed that the constraining effect of steel tubes on LAC was enhanced with the increase of steel area ratio. The confining pressure effect coefficient of LAC can be set to 3.4, which reflects the improvement of its stress performance under compression in the stroke direction. Zhou et al. [30] studied the effect of FRP constraint on the mechanical properties of LAC, and the results showed that the strength and ultimate deformation of LAC were greatly improved after being wrapped with FRP.

In order to reduce the weight of the structure, the combination of LAC with spiral stirrups, steel pipes and carbon fiber cloth is used in the engineering structure, which leads to the LAC being in a complex stress state. Therefore, it is very important to investigate the failure characteristics and mechanical properties of lightweight aggregate concrete under triaxial stress. For engineering purposes, 156 fly ash ceramsite aggregate concrete specimens were designed for conventional triaxial compression and uniaxial compression tests. Three factors, the soaking time of ceramsite aggregate, strength of concrete and confining pressure, were considered in the experiment to obtain the test data and to analyze the failure mechanism of the specimen and the stress-strain relationship in the process of compression. We hope that the experiment can provide support for the popularization and application of fly ash ceramsite aggregate concrete and promote a new idea for the recovery and recycling of fly ash.

## 2. Experimental Program

### 2.1. Materials

Samples of fly ash ceramsite coarse aggregate were used in this experiment. The particle sizes of coarse aggregate ranged from 2 to 10 mm with continuous gradation, and 93.86% of the particle sizes had continuous distribution (i.e., 5–8 mm). The saturated water absorption rate of the ceramsite was about 17.22%, and the soaking time to reach the saturated state was about 17 h. The water absorption of aggregate soaked for 1 h and 12 h could reach 86.88% and 93.61% of saturated water absorption, respectively. Table 1 shows the physical properties of coarse aggregates, including fly ash content, bulk density, numerical tube pressure, 1 h and 12 h of water absorption and saturated water absorption. The sieve allowance of fly ash was 18.4%. Ordinary Portland cement type 42.5 R was used as the binding material [31], and river sand was adopted as fine aggregate.

### 2.2. Mixing Proportion

The mixing proportions of LAC were determined according to JGJ/T 12-2019 [32], with three target compressive strengths of concrete (i.e., 20 MPa, 30 MPa, 40 MPa). The relevant data are shown in Table 2.

### 2.3. Specimen Fabrication

The objective of this experimental program was to study the behavior of LAC under triaxial compression conditions. A total of 156 cylindrical specimens were designed considering the aggregate immersion time (i.e., 1 h and 12 h), compressive strength of concrete and lateral confining pressure (i.e., restraint stress *σ*_2_, *σ*_3_) as the main research parameters of this experiment.

For the LAC tests, three concrete strengths (i.e., 20 MPa, 30 MPa, 40 MPa) were considered. During the test design, the commonly used concrete strength grade C30 was considered as the main research object, and the specimens of strength grade C20 and C40 were taken as the control group. In order to optimize the number of specimens, the control group was appropriately reduced. At the same time, considering that the peak stress (*σ*_1_) of C20 specimens reach the peak stress (*σ*_1_) level of C30 specimens under triaxial compression require greater confining pressure, and that of C40 specimens require less confining pressure. Therefore, compared with C30 specimens, the confining pressure range of C20 specimens is larger, and that of C40 specimens is smaller. On the other hand, the RMT-301 testing machine can provide a confining pressure value from 0 to 50 MPa. For the above reasons, for the LAC20 (20 MPa) tests, eight different levels of confinement stress were used in the range of *σ*_3_ = 0 to 42 MPa in 6 MPa increments; for the LAC30 (30 MPa) tests, twelve different levels of confinement stress were set in the range of *σ*_3_ = 0 to 33 MPa in 3 MPa increments; for the LAC40 (40 MPa) tests, six different levels of confinement stress were set in the range of *σ*_3_ = 0 to 15 MPa in 3 MPa increments. With regards to the nomenclature used for the specimens, LACf-m-n means that the specimen is LAC, the strength of concrete was f MPa, the aggregate was soaked for m hours and the confining pressure was n MPa. The specimen matrix is shown in Table 3.

The concrete was poured into custom-made circular molds with nominal dimensions of 100 mm in diameter and 200 mm in height, and then carefully mixed and vibrated. Before mixing the aggregates, the fly ash ceramsite was soaked for one or twelve hours, and then the water on the surface was drained. After 24 h, all samples were taken out of their molds and stored in a standard curing chamber at a relative humidity of 100% and a temperature of 20 ± 2 °C for 28 days.

It should be noted that the fly ash ceramsite aggregate had large porosity and could absorb large amounts of water. Once the concrete mixture was prepared, the fly ash ceramsite aggregate would continue to absorb water, which can be controlled by pre-wetting the aggregate. This also allows for the internal curing of concrete, as its water-bearing state can affect the hydration reaction and self-shrinkage potential of internal concrete. Accordingly, the influence of soaking time of fly ash aggregate is considered in this paper.

### 2.4. Mechanical Test Equipment

**Figure 1:**
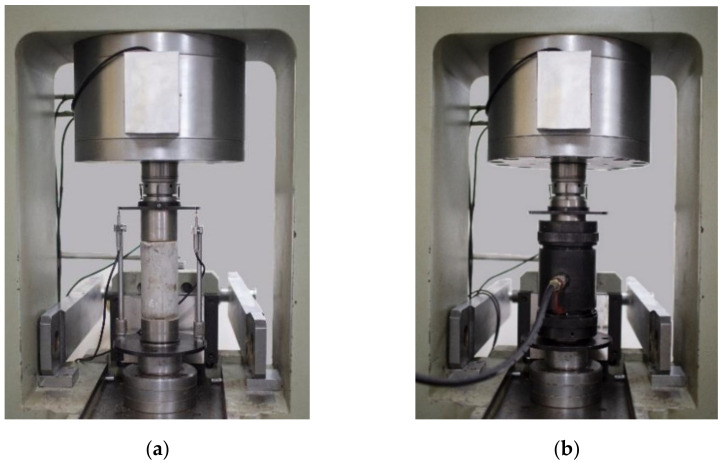
Test setup. (**a**) Uniaxial compression tests. (**b**) Triaxial compression tests.

The uniaxial and triaxial compression tests were conducted on an RMT-301 test system that can realize the axisymmetric triaxial experiments. The RMT-301 test system was developed by CAS Wuhan Institute of Rock and Soil Mechanics. The vertical hydraulic cylinder was equipped with two different range force sensors and displacement sensors. Figure 1 shows the loading device for uniaxial and triaxial compression tests.

**Figure 2:**
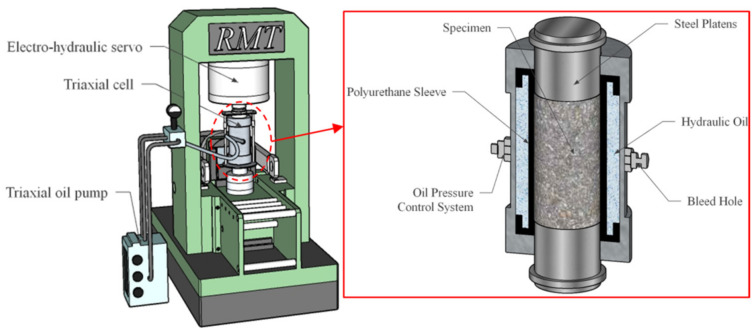
The pressure vessel assembly for the triaxial test.

**Figure 3:**
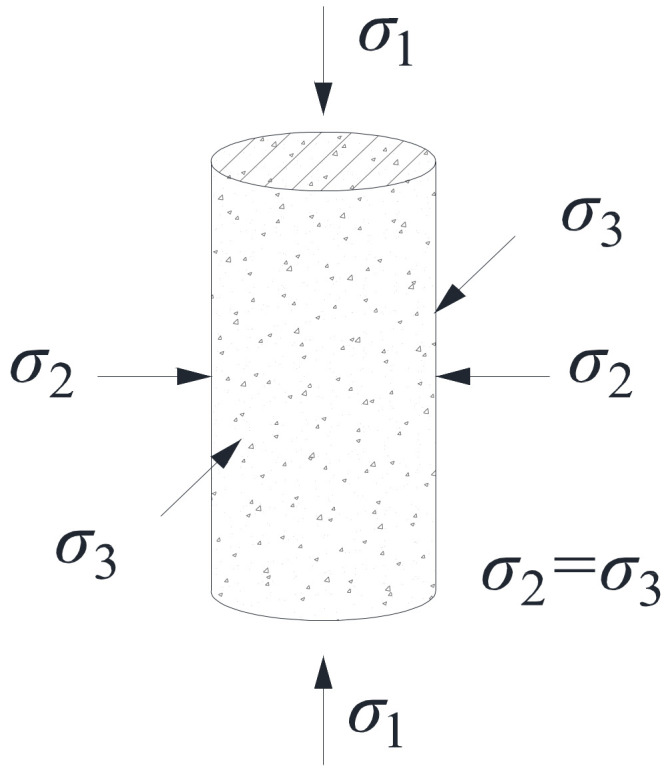
Triaxial stress model of specimen.

Figure 2 shows the isolated pressure chamber assembly for the triaxial test. During triaxial compression tests, a stretchable polyurethane isolation sheath was used to prevent hydraulic oil from penetrating the sample during triaxial compression. The triaxial compression model of the specimen in the device is shown in Figure 3. Where principal stresses *σ*_2_ = *σ*_3_, and are constant.

### 2.5. Loading Protocol

**Figure 4:**
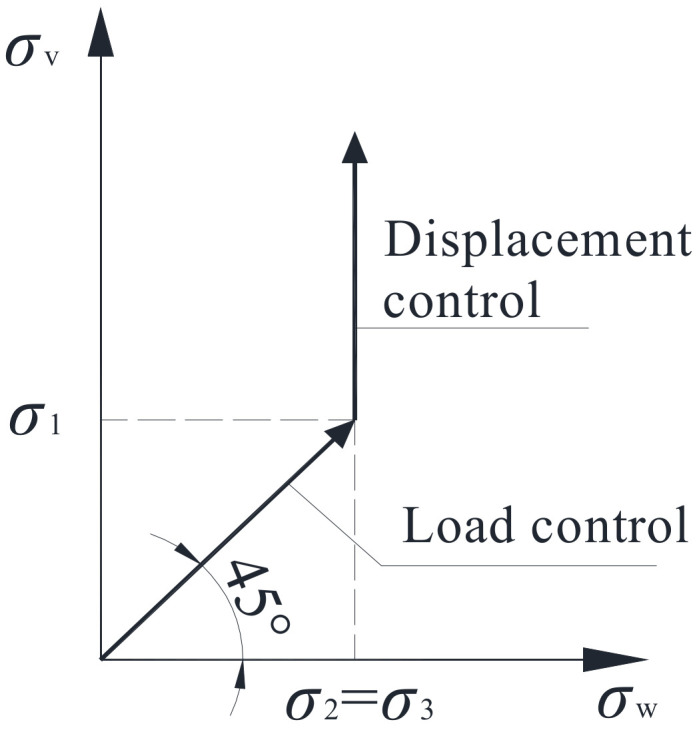
Loading process.

Figure 4 shows the triaxial compression test loading mode with the mixed control of load and displacement. First, load control was used, then displacement control was used. In the load control stage, when the preset confining pressure *σ*_2_ = *σ*_3_ was applied, the axial stress *σ*_1_ was applied at constant speed until the specimen was in the triaxial hydrostatic stress state (i.e., *σ*_1_ = *σ*_2_ = *σ*_3_). At this moment, the confining pressure was kept constant, and the vertical load adopted displacement control with the loading rate of 0.01 mm/s until the specimen failed. The uniaxial compression test adopted displacement control of the loading system, and the axial loading rate was 0.01 mm/s.

## 3. Results and Discussion

### 3.1. Failure Modes

**Figure 5:**
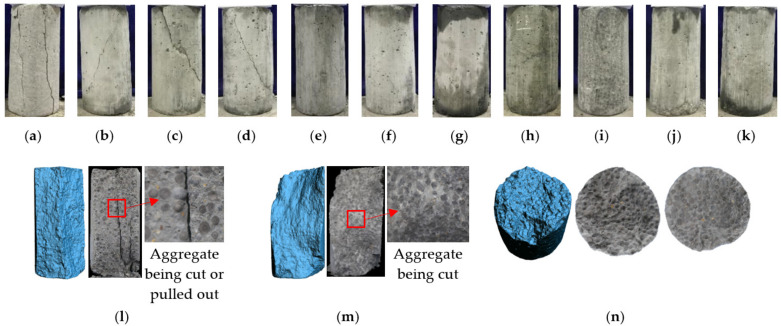
The typical cracking patterns. (**a**) 0 MPa, (**b**) 3 MPa, (**c**) 6 MPa, (**d**) 9 MPa, (**e**) 12 MPa, (**f**) 15 MPa, (**g**) 18 MPa, (**h**) 21 MPa, (**i**) 24 MPa, (**j**) 27 MPa, (**k**) 30 MPa, (**l**) uniaxial compression failure modes, (**m**) oblique-shear failure pattern (*σ*_w_ = 6 MPa), (**n**) oblique-shear failure pattern (*σ*_w_ = 30 MPa).

The typical cracking patterns and failure modes of LAC (LAC30 specimens) under uniaxial (*σ*_2_ = *σ*_3_ = 0) and triaxial (*σ*_2_ = *σ*_3_ > 0) compression tests are presented in Figure 5. It was observed that the confining stress had a great influence on the failure mode of the specimen, while the effects of concrete strength and aggregate soaking time are less pronounced.

In the case of uniaxial (*σ*_2_ = *σ*_3_ = 0) compression failure modes, with the increase of test load, a vertical crack appears in the middle of the specimens at first, and then several vertical cracks start to grow and rapidly extend to both ends of the specimen (Figure 5a). After reaching the peak load, the bearing capacity decreases rapidly, and the specimens appear as columnar (or vertical splitting) failures.

In the case of triaxial (3 MPa ≤ *σ*_2_ = *σ*_3_ ≤ 12 MPa) compression failure modes, as shown in Figure 5b–e, oblique shear failure was observed with oblique cracks running through the specimen to form the upper and lower halves. The angle between the main crack and the cross-section was around 50–70°, and this angle decreased with the increasing confining pressure.

In the case of triaxial (15 MPa ≤ *σ*_2_ = *σ*_3_ ≤ 33 MPa) compression failure modes, it can be seen in Figure 5f–k that the specimen underwent significant compression deformation in the A-axis direction, and no obvious cracks appeared on the surface. However, the originally exposed holes on the specimen surface were deformed under pressure, and the middle part was laterally warped. When tapping the middle part of the specimen, the specimen broke in half along the lateral direction in the middle, indicating that many horizontal cracks had been formed in the specimen.

Figure 5l–n shows the failure surfaces of LAC (LAC30 specimens) under uniaxial and triaxial compression tests. It can be seen in the figure that the fly ash ceramsite aggregates were partially intact under uniaxial compression. Under triaxial stress, the ceramsite aggregate in the failure section of the specimen was shear damaged under low confining pressure, and horizontal cracks appeared in the failure section of the specimen under high confining pressure.

### 3.2. Stress-Strain Behavior

**Figure 6:**
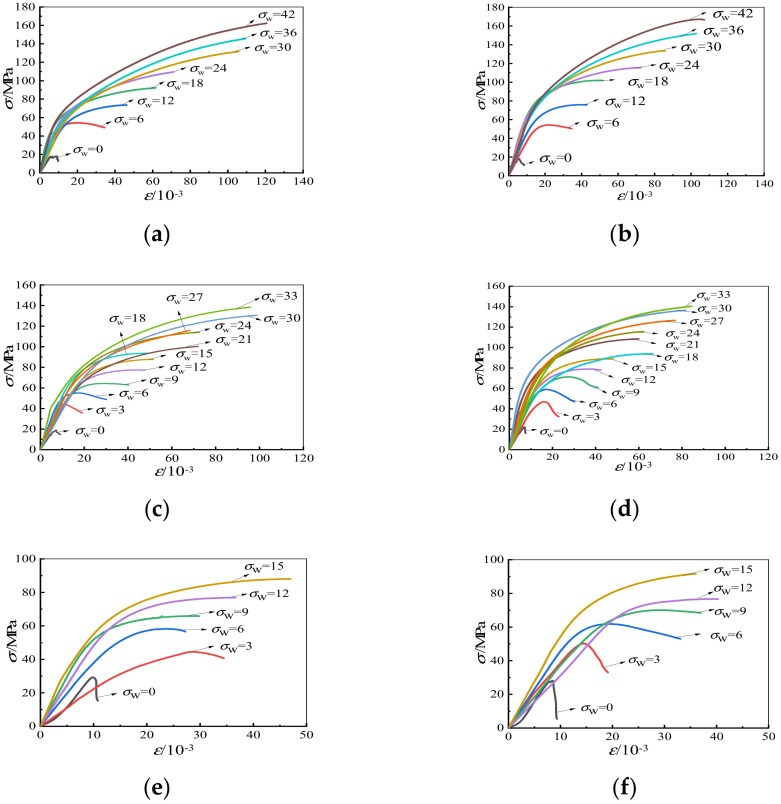
The stress-strain curves. (**a**) LAC20, Soak for 1 h, (**b**) LAC20, Soak for 12 h, (**c**) LAC30, Soak for 1 h, (**d**) LAC30, Soak for 12 h, (**e**) LAC40, Soak for 1 h, (**f**) LAC40, Soak for 12 h.

Figure 6 shows stress-strain curves of all specimens, and Table 4 shows compressive strength values.

From Figure 6, it can be inferred that the shape of stress-strain curves is greatly influenced by the lateral confining pressure. The relationship between stress and strain is almost linear at a lower axial stress state, but nonlinear at a high-stress state. The descending branch of the triaxial compression stress-strain curves becomes smoother (i.e., higher ductility), and when the confining stress reaches 15 MPa (*σ*_3_ = *σ*_2_ = 15 MPa), the stress-strain curve continues to rise. The low-strength concrete specimens have greater compressive deformation capacity under triaxial stress, and the aggregate immersion time has little effect on the stress-strain curves of specimens.

### 3.3. Influence of Concrete Strength

**Figure 7:**
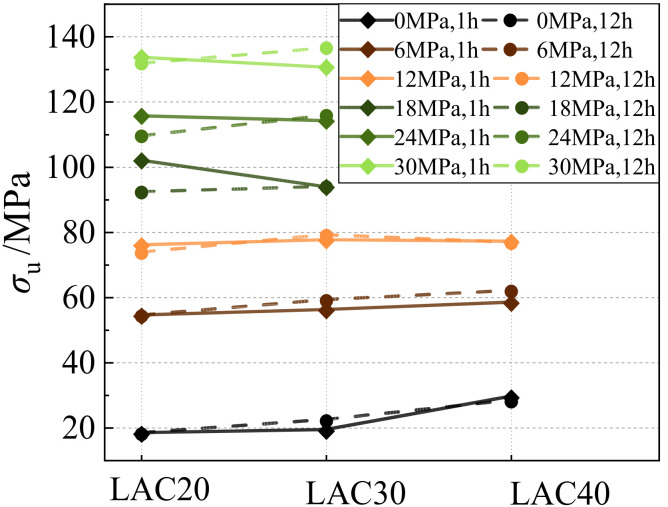
Relationship between concrete strength grade and peak stress.

Figure 7 demonstrates that the peak stress of LAC increases with the growth of concrete strength under uniaxial compression. However, the value of peak stress is slightly affected by the strength of concrete under triaxial compression. At the same confining pressure condition, the average fluctuation of peak stress caused by the strength change of lightweight aggregate concrete was about 1.8%. This is because the compressive strain generated by the lateral confining pressure on concrete can prevent the tensile strain generated by the vertical load to a certain extent, such that aggregate and cement matrix are no longer the main factors that bear the load action, indicating that the existence of confining pressure can weaken the influence of strength grade on peak stress.

### 3.4. Influence of Confining Pressure

**Figure 8:**
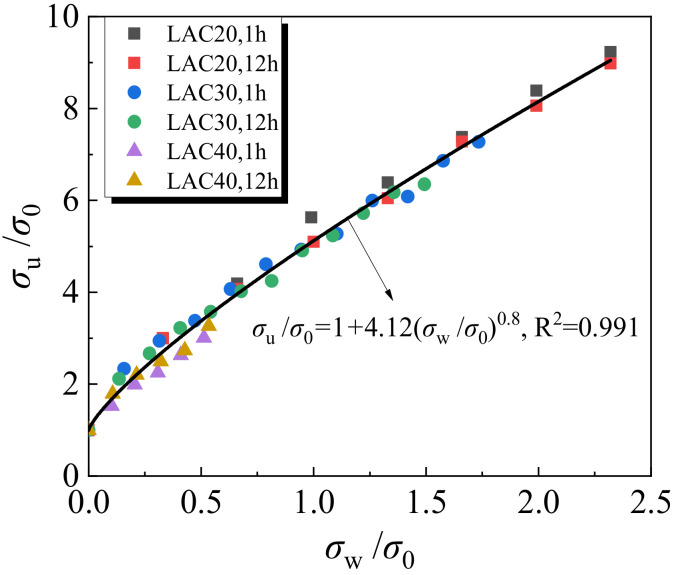
Relationship between compressive strength and lateral confining pressure.

The relationship between compressive strength and confining pressure of lightweight aggregate concrete is shown in Figure 8. It can be seen that the value of confining pressure (*σ*_w_) has a significant impact on the peak stress (*σ*_u_) under triaxial stress, and the peak stress (*σ*_u_) increases with the rise of lateral restraint stress (*σ*_w_). Through nonlinear regression analysis, the relation equation between *σ*_u_ and *σ*_w_ is proposed as follows:

(1) $$\frac{\sigma_{u}}{\sigma_{0}}=1+4.12{\left(\frac{\sigma_{w}}{\sigma_{0}}\right)}^{0.8}$$

where *σ*_w_ denotes the lateral confining pressure, *σ*_w_ = *σ*_2_ = *σ*_3_, *σ*_u_ denotes the corresponding axial peak stress, *σ*_u_ = *σ*_1_ and *σ*_0_ denotes the uniaxial compressive strength of concrete.

### 3.5. Influence of Aggregate Immersion Time

**Figure 9:**
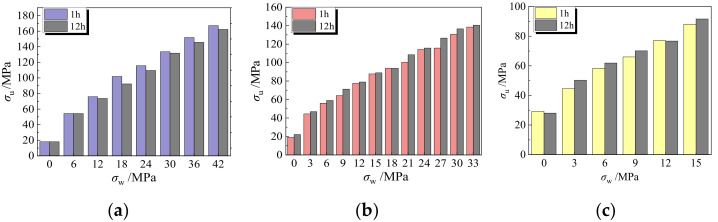
The influence of soaking time under the same confining pressure. (**a**) LAC20, (**b**) LAC30, (**c**) LAC40.

Figure 9 shows the variation of peak stress with confining pressure for LAC with an aggregate immersion time of 1 h and 12 h. According to Figure 9a, when the lateral confining pressure *σ*_w_ ≤ 6 MPa, the peak stress of LAC (LAC20) was hardly affected by the immersion time of the aggregate. However, when the lateral confining pressure was greater than 6 MPa, the peak stress of LAC in which the aggregate had been soaked for 12 h was about 5% smaller on average than that in which the aggregate was soaked for 1 h. As seen in Figure 9b–c, for LAC with design strengths of 30 MPa and 40 MPa, the peak stress of fly ash ceramsite aggregate soaked for 12 h was greater than that soaked for 1 h, which constitute an increase of approximately 5.0% and 3.7%, respectively.

The reason for the above phenomenon may be that the longer the immersion time of fly ash ceramsite aggregate, the more water is absorbed in its pores, which provides the secondary hydration reaction condition of the aggregate interface during the concrete curing process, improving the structural compactness of the interface [19] and strengthening the fly ash ceramsite concrete (LAC30 and LAC40). On the other hand, the water:cement ratio of LAC20 is relatively large, and too much water in the void of the fly ash ceramsite aggregate lowers the strength of LAC.

### 3.6. Failure Mechanism Analysis

#### 3.6.1. Failure Mechanism under Uniaxial Compression

**Figure 10:**
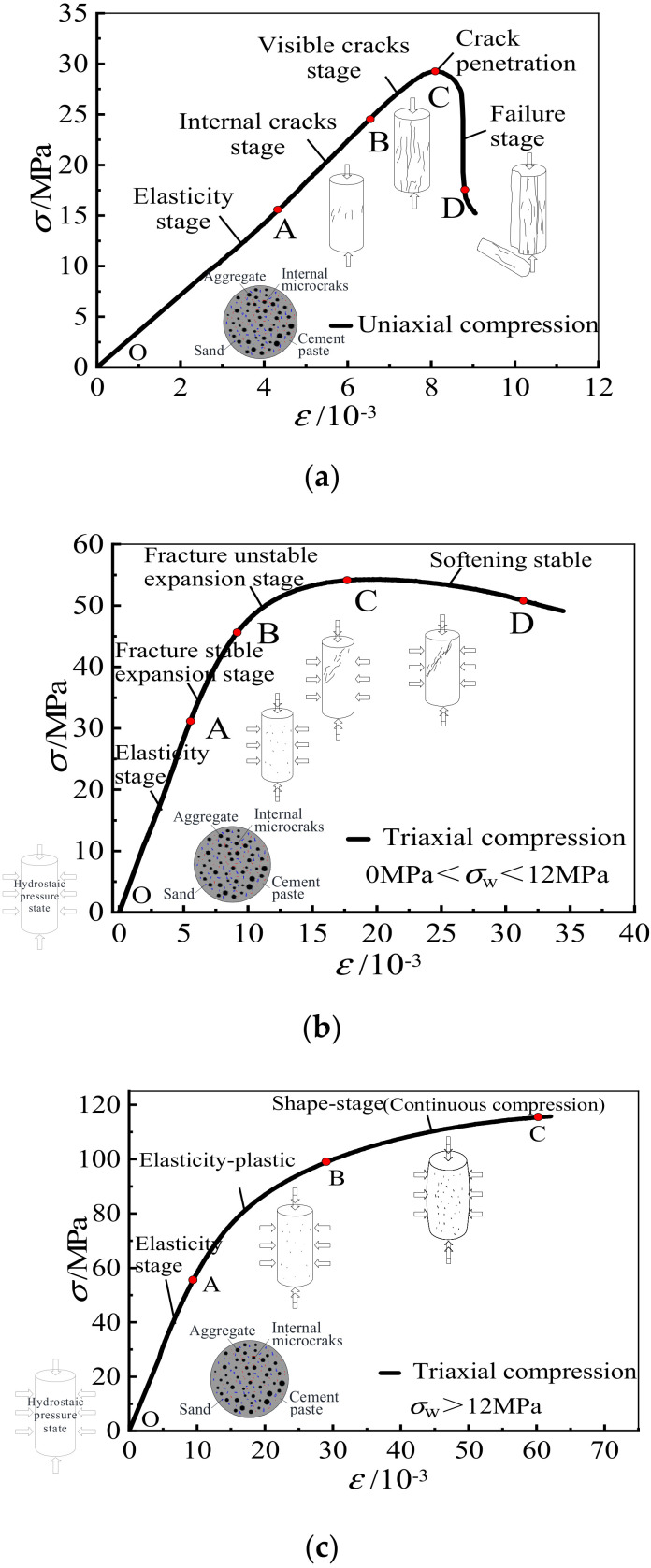
Failure process of specimens. (**a**) Failure process of stress-strain curve under uniaxial compression. (**b**) Failure process of stress-strain curve under triaxial compression (0 MPa < *σ*_w_ ≤ 12 MPa). (**c**) Failure process of stress-strain curve under triaxial compression (*σ*_w_ > 12 MPa).

Figure 10a illustrates the failure process of fly ash ceramsite LAC under uniaxial compression. The specimens suffered vertical columnar splitting failure after five stages: elastic deformation stage (OA), internal crack development stage (AB), visible crack development stage (BC) and failure stage (CD). The stress and strain have an obvious linear relationship when the actual (specimen) stress is less than 40~50% of the peak stress. The LAC enters the period of continuous crack development with the increase of load, and at this stage, the lateral tensile strain exceeds the ultimate tensile strain of the lightweight aggregate concrete and microcracks appear on the surface of the specimens. When the specimen stress is close to the peak stress, the crack expands and increases rapidly. When the specimen stress exceeds the peak stress, cracks run through and form open cracks; vertical split failure occurs in the LAC and part of the ceramsite aggregate on the failure surface is cut off.

#### 3.6.2. Failure Mechanism under Triaxial Compression

When the confining pressure *σ*_w_ ≤ 12 MPa, the triaxial compression specimen was in a state of hydrostatic stress in the load control stage. With the increase of axial stress, the lateral strain gradually changed from compressive strain to tensile strain due to the Poisson effect. Figure 10b shows the failure process of fly ash ceramsite lightweight aggregate concrete under triaxial compression. In the elastic stage (OA section), the load and displacement linearly increase. With the increase of vertical load, the fly ash ceramsite aggregate and mortar both have a certain degree of extrusion deformation, and small cracks begin to appear on the surface of the specimens (AB section). With the increase of axial compressive stress, the cracks extend rapidly through the mortar and aggregate forming obvious penetrating oblique cracks, and almost all of the lightweight aggregate in the failure section is shear damaged (BC section). However, due to the presence of confining pressure, the specimens show oblique shear failure, and the oblique shear angle decreases with the increase of confining pressure. Finally, the lightweight aggregate concrete comes to be in a weak state (CD section). In this stage, the larger the lateral confining pressure, the greater the degree of compression deformation, and the gentler the vertical stress decline.

However, when *σ*_w_ ≥ 12 MPa, the compression strain caused by the confining pressure limited the development of tensile strain formed by the axial load. Figure 10c shows the failure process. In the elastic stage (OA section), the load and displacement linearly increased, and initial microcracks appeared in the concrete. After reaching the elastic-plastic stage (AB section), the crack could not develop to the surface due to the large confining pressure; however, the axial deformation increased.

### 3.7. Microstructure of LAC

**Figure 11:**
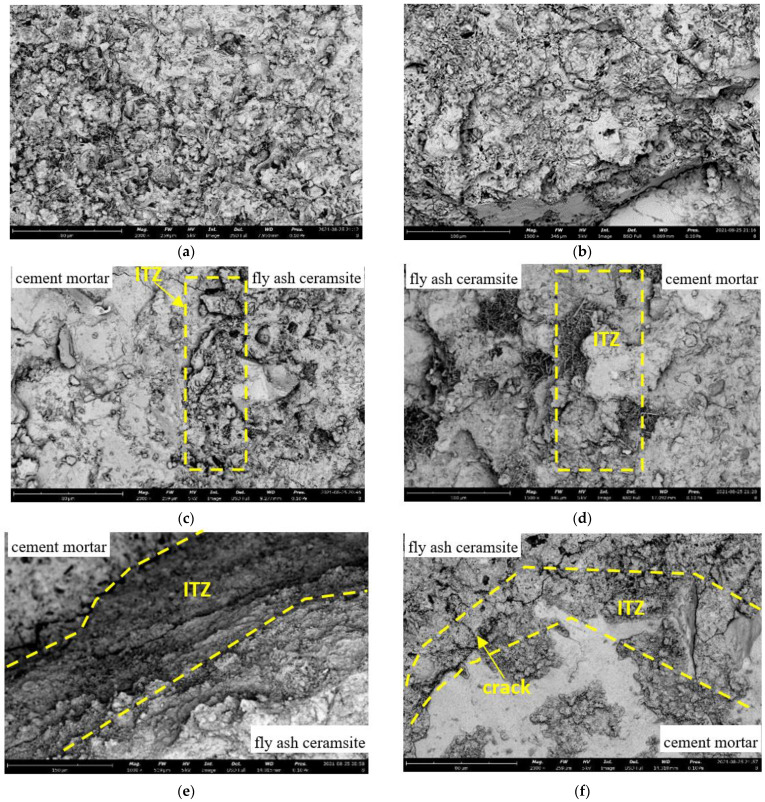
The SEM micrographs of ceramsite aggregate concrete. (**a**) Ceramsite aggregate surface (LAC30-1-0), (**b**) cement mortar (LAC30-1-0), (**c**) the interface between ceramsite aggregate and cement mortar (LAC30-1-0). (**d**) The interface between ceramsite aggregate cross-section and cement mortar (LAC30-1-6). (**e**) The interface between ceramsite aggregate and cement mortar (LAC30-12-6). (**f**) The interface between ceramsite aggregate cross-section and cement mortar (LAC30-1-30).

The microstructures of the LAC sample after loading are presented in Figure 11. As shown in the figure, the fly ash ceramsite microstructure has many pores and floccules (Figure 11a), which is the reason for the low weight and high-water absorption of ceramsite aggregate. However, the cement mortar shown in Figure 11b has a relatively dense structure with lower porosity and irregular texture. By comparing Figure 11c,f, it can be established that, after loading the specimen with 30 MPa confining pressure, there are more microcracks inside the material, and cracks also appear at the interface between ceramsite and cement mortar. Figure 11d,e presents the SEM micrographs of the interfacial transition zone (ITZ) between aggregates and cement paste, with the ceramsite aggregate soaked for 1 h and 12 h, respectively. Once the aggregates have been soaked for 12 h, there are fewer pores and floccules at the interface between the aggregate and cement slurry, and the hydration reaction at the interface is more sufficient.

## 4. Failure Criteria for LAC

Previous research shows that the strength criteria can be generally divided into three categories. The first is the empirical model, such as the Willam-Wamke model and the Kotsovos-Pavlovic model. The second type is based on the physical and mechanical properties of concrete, such as the Mohr–Coulomb model. The third type is based on the analysis of experimental results, such as the Ottosen four-parameter model. These concrete strength criteria reveal the law of failure under a complex stress state and provide guidance for the engineering structure design, which has great significance. Based on the test data and the existing strength theory, this paper discusses the failure criterion of LAC under the conventional triaxial condition from a macroscopic point of view.

### 4.1. Mohr–Coulomb Criterion

**Figure 12:**
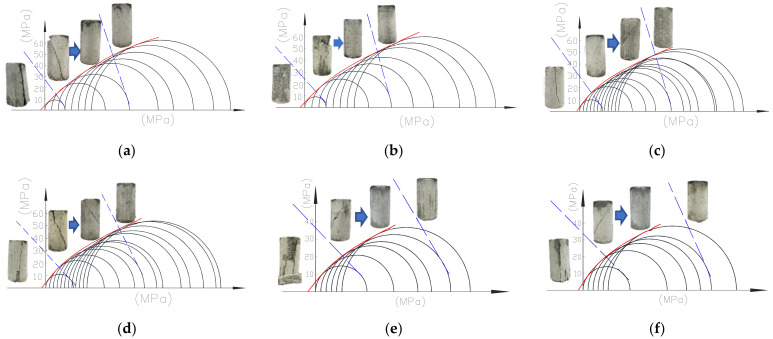
Mohr–Coulomb stress envelope for LAC. (**a**) LAC20, soak for 1 h; (**b**) LAC20, soak for 12 h; (**c**) LAC30, soak for 1 h; (**d**) LAC30, soak for 12 h; (**e**) LAC40, soak for 1 h; (**f**) LAC40, soak for 12 h.

The Mohr–Coulomb criterion states that failure is governed by the relation where the limiting shearing stress *τ* in a plane is dependent only on the normal stress *σ* in the same plane at one point. The Mohr circle stress envelope defines the failure stress state of the specimen. According to the Mohr–Coulomb criterion, the failure of LAC will occur for all states of stress for which the Mohr circles are exactly tangent to the envelope. Based on the test data, the Mohr stress circles and Mohr circle envelope of LAC under different confining pressures are drawn in Figure 12. Figure 12a–f respectively shows the stress circles of LAC20, LAC30 and LAC40 strength, with the light aggregate soaked for 1 h and 12 h. At the same time, the failure mode of confining pressure corresponding to the stress circle is given in the figure.

As shown in Figure 12, the envelope curve is a power function. Through data fitting, the following failure envelope equation is obtained as:

(2) $$\tau_{u}=c+\mu \sigma_{u}^{a}$$

where *τ*_u_ and *σ*_u_ are respectively the shear and normal stress, *c* represents the “cohesion” for LAC that is the intersection of the Mohr circle envelope and vertical axis, *μ* and a are the curve fitting parameters and *μ* and a are the fitting coefficients.

**Figure 13:**
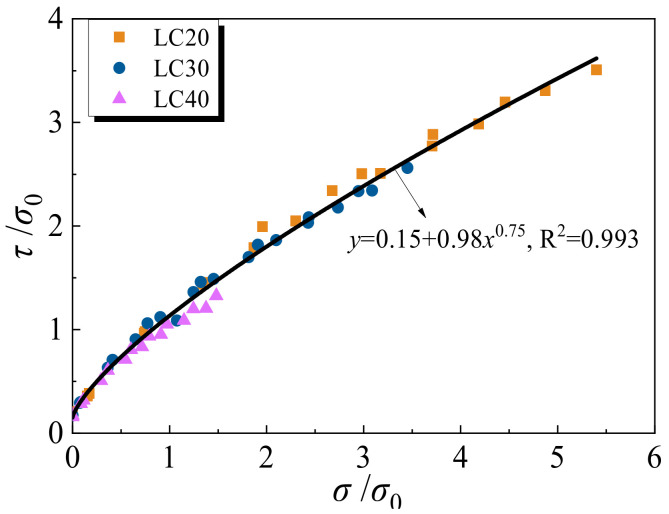
Normalized *τ*-*σ* diagram.

The shear stress and normal stress in the equation are normalized for application consideration. The normalized results are shown in Equation (3):

(3) $$\frac{\tau_{u}}{\sigma_{0}}=\varphi +\gamma {\left(\frac{\sigma_{u}}{\sigma_{0}}\right)}^{a}$$

where $\varphi =\frac{c}{\sigma_{0}}$; and $\gamma =\mu \sigma_{0}^{a-1}$. The values of *a*, *φ* and *γ* can be obtained by fitting as follows: *a* is 0.75, *φ* is 0.15, *γ* is 0.98, *σ*_0_ is uniaxial compressive strength. The fitting curve is shown in Figure 13.

### 4.2. Shear Stress Failure Criterion

The stress in any point of LAC is expressed by three principal stresses *σ*_1_, *σ*_2_ and *σ*_3_ as three axes in the Cartesian coordinate system, and an octahedral principal stress space can be obtained where the hydrostatic pressure line represents the stress state *σ*_1_ = *σ*_1_ = *σ*_3_. The plane perpendicular to the hydrostatic pressure line is the octahedral principal stress plane, and its normal stress and shear stress τ are expressed by the formula:

(4) $$\tau =\frac{1}{3}\sqrt{{\left(\sigma_{1}-\sigma_{2}\right)}^{2}+{\left(\sigma_{2}-\sigma_{3}\right)}^{2}+{\left(\sigma_{1}-\sigma_{3}\right)}^{2}}$$

(5) $$\bar{\sigma }=\frac{1}{3}\left(\sigma_{1}+\sigma_{2}+\sigma_{3}\right)$$

The test data were input into Equations (4) and (5) and normalized with *σ*_0_ (the uniaxial compressive strength). The normalized shear stress failure model obtained through the fitting analysis is as follows:

(6) $$\frac{\tau }{\sigma_{0}}=1.97{\left(\frac{\bar{\sigma }}{\sigma_{0}}\right)}^{0.82}$$

**Figure 14:**
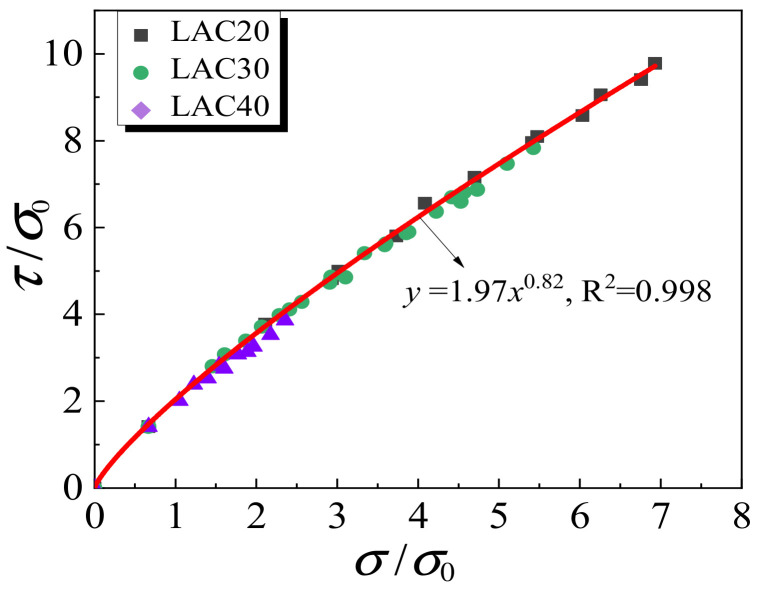
Normalized *τ*-*σ* curves.

Equation (6) is only applicable to the stress state of the experiment in this paper. It can be seen in Figure 14 that *τ* and *σ* can be precisely related by using the proposed equation under the stress conditions of this experiment.

### 4.3. Compression Meridian Plane Model

**Figure 15:**
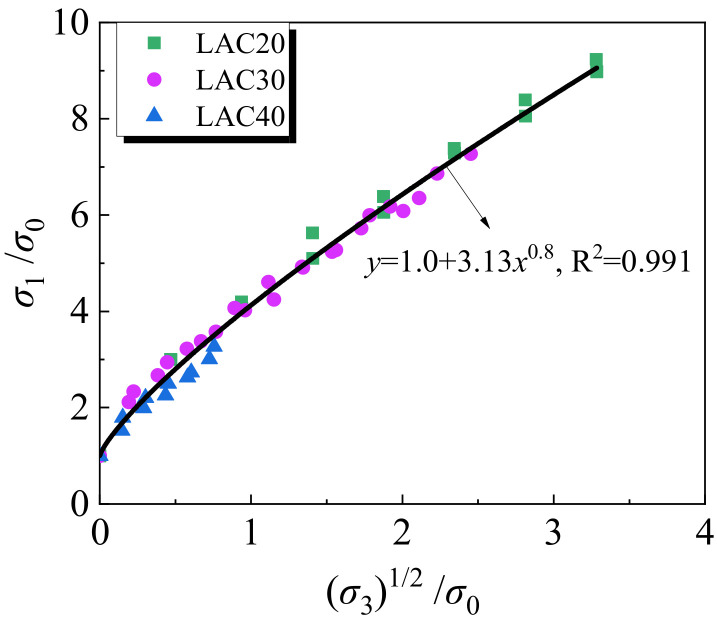
Normalized *σ*_1_-$\sqrt{2}$*σ*_3_ curves.

Under the conventional triaxial condition (*σ*_1_ > *σ*_2_ = *σ*_3_), the measured results were drawn as the triaxial compression failure surface of LAC. This is a plane with a principal stress axis and a diagonal in space, also called the Rendulic plane or the pressure meridian plane. The measured results are summarized, and *σ*_u_ and $\sqrt{2}$*σ*_3_ are dimensionless parameters; the relation equation of *σ*_u_ and $\sqrt{2}$*σ*_3_ can be written as follows:

(7) $$\frac{\sigma_{u}}{\sigma_{0}}=1.0+\varsigma {\left(\frac{\sqrt{2}\sigma_{3}}{\sigma_{0}}\right)}^{\phi }$$

where *ζ* and *Φ* are fitting constants. Through fitting analysis, *ζ* and *Φ* of LAC are 3.13 and 0.8, respectively. The comparison of Equation (7) with the test data for LAC is shown in Figure 15.

The above three failure criteria can well simulate the failure curve of fly ash ceramsite aggregate concrete. Due to the large internal structural porosity of fly ash ceramsite, its aggregate strength is far less than that of natural aggregate; coarse aggregate has a great influence on the mechanical performance of concrete, which often features stress concentration in the aggregate, aggregate and cementing interface, resulting in cracking. In addition, the density of fly ash ceramsite is smaller than that of cement slurry, and it will easily float or segregate in the process of stirring and vibrating, which will also affect the working performance of fly ash ceramsite LAC. Therefore, under the action of triaxial stress, both the aggregate and the cement matrix will be shear damaged. The angle between the failure surface and the direction of principal stress is not consistent with the direction of maximum shear stress when LAC is destroyed under multi-axial stress. Among the failure criteria, the Mohr–Coulomb theory is considered from the point of view that the friction force in concrete leads to its destruction, which can better reflect the destruction of fly ash ceramsite LAC under the triaxial stress state.

## 5. Stress-Strain Constitutive Relationship Model

### 5.1. Uniaxial Stress-Strain Constitutive Relationship

**Figure 16:**
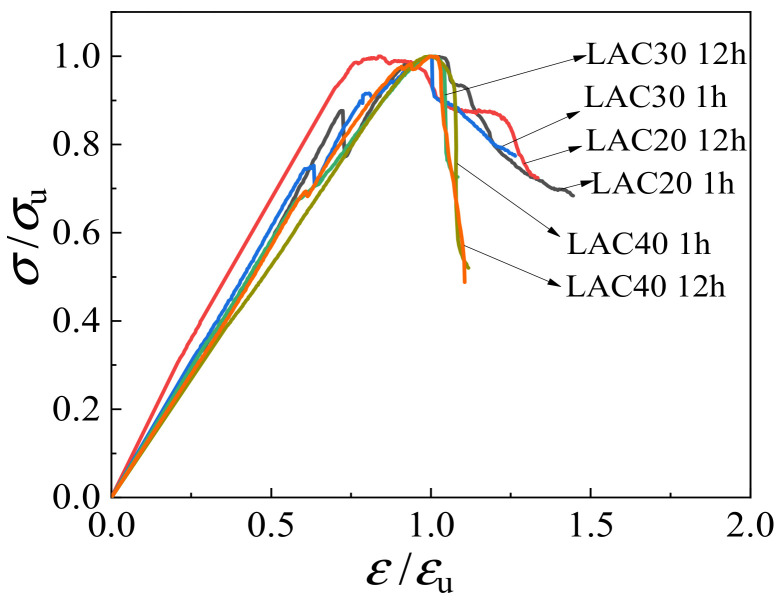
Uniaxial dimensionless stress-strain curves.

Figure 16 depicts the relationships between axial stress *σ*/*σ*_u_ and strain *ε*/*ε*_u_ of LAC. The figure shows that the ascending branch of the curve is obviously affected by the intensity grade. There is a big difference in the downward part of the curve, which shows that the higher the strength grade and the longer the soaking time of the aggregate, the steeper the drop in the curve.

**Figure 17:**
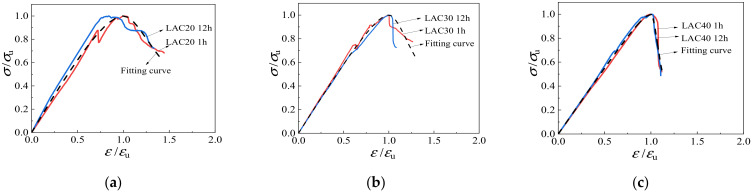
Comparison of uniaxial compression test curve and fitting curve. (**a**) LAC20, (**b**) LAC30, (**c**) LAC40.

Considering the practical application, this paper adopted the analytic formula proposed by the Euro Code CEB-FIP (1990) [33] to describe the upward branch of the stress-strain curves, and adopted the analytic formula proposed by Guo [34] to represent the downward branch of the stress-strain curves. The fitted piecewise constitutive equation of LAC under uniaxial stress is as follows:

(8) $$y=\left\{\begin{array}{ll} \frac{kx-x^{2}}{1+(k-2)x} & 0\leq x<1\\ \frac{x}{q{\left(x-1\right)}^{2}+x} & x\geq 1 \end{array}\right.$$

where *x* = *ε*/*ε*_u_, *y* = *σ*/*σ*_u_, and *k* and *q* are the fitting coefficients. According to the data fitting analysis, the coefficients *k* and *q* of LAC20 were 1.32 and 4.82, respectively; the coefficients *k* and *q* of LAC30 were 1.20 and 8.28, respectively; and the coefficients *k* and *q* of LAC40 were 1.11 and 70.67, respectively. The comparison between the fitting results and the curve under different parameters is shown in Figure 17. The proposed stress-strain equation can match the experimental results of LAC well.

### 5.2. Triaxial Compression Stress-Strain Constitutive Relationship

**Figure 18:**
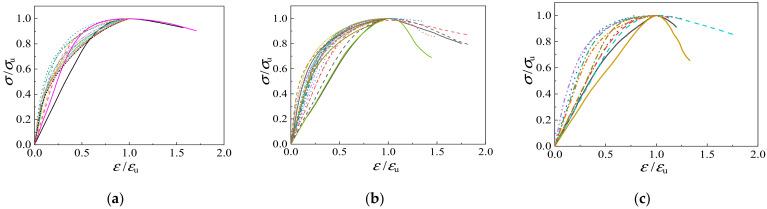
Triaxial dimensionless stress-strain curves. (**a**) LAC20, (**b**) LAC30, (**c**) LAC40.

Figure 18 presents the relationships between axial stress *σ*/*σ*_u_ and strain *ε*/*ε*_u_ under triaxial compression of light aggregate concrete. The stress-strain curves are not affected by the soaking time of the aggregate, but are greatly influenced by the strength grade and the lateral confining pressure value. Firstly, the strength grade of fly ash ceramsite concrete has no significant influence on the rising part of the curve, but has a certain influence on the declining part. The higher the strength grade, the steeper the drop in the curve. Secondly, with the increase of confining pressure, the initial elastic modulus of LAC under triaxial compression becomes larger, the ascending part of the curve gets steeper and the descending part becomes smoother. However, when the confining pressure is greater than 12 MPa, the descending section basically does not appear in the curve.

**Figure 19:**
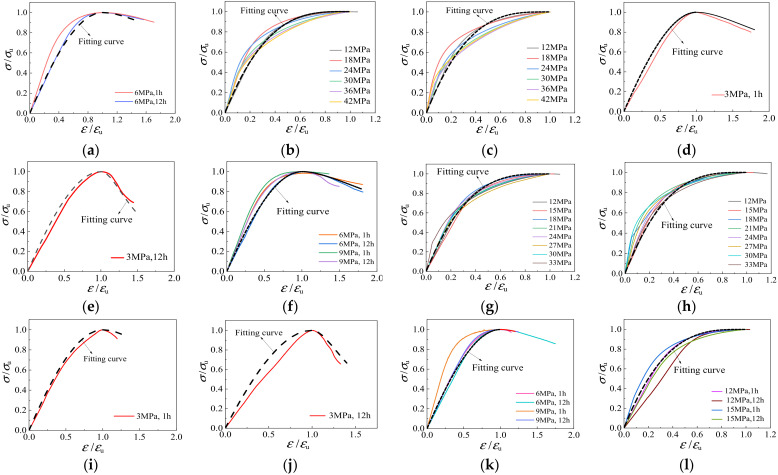
Comparison of test curve and fitting curve under different parameters. (**a**) LAC20, 0 MPa < *σ*_w_ ≤ 6 MPa; (**b**) LAC20, soak for 1 h, *σ*_w_ > 6 MPa; (**c**) LAC20, soak for 12 h, *σ*_w_ > 6 MPa; (**d**) LAC30, soak for 1 h, *σ*_w_ ≤ 3 MPa; (**e**) LAC30, soak for 12 h, *σ*_w_ ≤ 3 MPa; (**f**) LAC30, 3 MPa < *σ*_w_ ≤ 9 MPa; (**g**) LAC30, soak for 1 h, *σ*_w_ > 9 MPa; (**h**) LAC30, soak for 12 h, *σ*_w_ > 9 MPa; (**i**) LAC40, soak for 1 h, *σ*_w_ ≤ 3 MPa; (**j**) LAC40, soak for 12 h, *σ*_w_ ≤ 3 MPa; (**k**) LAC40, 3 MPa < *σ*_w_ ≤ 9 MP; (**l**) LAC40, *σ*_w_ > 9 MP.

Since the stress-strain curve of LAC is similar in shape to that of NAC, the analytical formula proposed by Guo [34], which has been adopted by GB50010-2010 [35], is used to describe the stress-strain relationship of LAC under triaxial compression. The stress-strain relationship in triaxial compression is proposed as follows:

(9) $$y=\left\{\begin{array}{ll} \lambda x+(3-2\lambda )x^{2}+(\lambda -2)x^{3} & 0\leq x<1\\ \frac{x}{\xi {\left(x-1\right)}^{2}+x} & x\geq 1 \end{array}\right.$$

where *x* = *ε*/*ε*_u_, *y* = *σ*/*σ*_u_ and *λ* and *ξ* are the fitting coefficients. In the ascending and descending stages, the coefficients of confining pressure *σ*_w_ ≤ 6 MPa and confining pressure *σ*_w_ > 6 MPa, respectively, were fitted. The fitting results of coefficients *λ* and *ξ* are shown in Table 5 below. The comparison between the fitting results and the curve under different parameters is shown in Figure 19. The proposed stress-strain equation can match the experimental results of LAC well.

## 6. Conclusions

This paper introduces the test results for the mechanical properties and failure behavior of fly ash ceramsite concrete (lightweight aggregate concrete, LAC) under uniaxial and triaxial compression. The findings of the study can be summarized as:

1. The stress-strain curve of LAC under triaxial compression is affected more by the strength grade and lateral confining pressure value and slightly by the soaking time of the aggregate. When the confining pressure is 0 MPa < *σ*_w_ ≤ 12 MPa, the stress-strain curve has a descending branch. Meanwhile, there is an obvious stress plateau in the stress-strain curve when the confining pressure is *σ*_w_ > 12 MPa.
2. The value of peak stress is slightly affected by the soaking time of aggregates in LAC. The variable range of peak stress is between −5% and 5% when the soaking time of aggregates increases from 1 h to 12 h.
3. There are three different failure mechanisms of LAC under continuous stress. First, the lateral tensile stress caused by vertical load leads to failure under uniaxial compression. Second, in the range of 0 MPa < *σ*_w_ ≤ 12 MPa, the oblique shear failure is caused by the joint action of lateral tensile stress and confining pressure binding. Third, when the confining pressure *σ*_w_ > 12 MPa, there are no visible cracks on the surface of the specimens, and the specimens are continuously pressed and compacted to form the central bulge failure.
4. The Mohr–Coulomb theory, octahedral shear stress theory and Rendulic plane theory were applied to analyze the failure criterion of LAC, and the applicable triaxial force calculation formula was obtained by fitting the dimensionless test data.
5. Referring to the segmental constitutive model of ordinary concrete proposed by Guo, the triaxial stress-strain constitutive equation of fly ash ceramsite LAC is proposed, and the theoretical results are in good agreement with the experimental results.

## Figures and Tables

**Table 1:** Physical properties of ceramsite aggregate.

Type	Fly Ash Content	Bulk Density (kg/m^3^)	Numerical Tube Pressure (MPa)	1 h Water Absorption	12 h Water Absorption	Saturated Water Absorption
Fly ash ceramsite	80%	650	7.2	14.96%	16.12%	17.22%

**Table 2:** Mix proportion of lightweight aggregate concrete.

Concrete Strength	Cement (kg/m^3^)	Ceramsite (kg/m^3^)	Sand (kg/m^3^)	Fly Ash (kg/m^3^)	Water (kg/m^3^)	Water-Cement Ratio
LAC20	350	420.3	667.4	75	170	0.40
LAC30	400	407.7	634.3	47.4	170	0.38
LAC40	450	392	609.7	22.2	170	0.36

**Table 3:** Parameters of specimens.

Specimen ID	Concrete Strength (MPa)	Soaking Time (h)	Confining Pressure (Mpa)	Specimen ID	Concrete Strength (Mpa)	Soaking Time (h)	Confining Pressure (Mpa)
LAC20-1-0	20	1	0	LAC30-1-30	30	1	30
LAC20-1-6	20	1	6	LAC30-1-33	30	1	33
LAC20-1-12	20	1	12	LAC30-12-0	30	12	0
LAC20-1-18	20	1	18	LAC30-12-3	30	12	3
LAC20-1-24	20	1	24	LAC30-12-6	30	12	6
LAC20-1-30	20	1	30	LAC30-12-9	30	12	9
LAC20-1-36	20	1	36	LAC30-12-12	30	12	12
LAC20-1-42	20	1	42	LAC30-12-15	30	12	15
LAC20-12-0	20	12	0	LAC30-12-18	30	12	18
LAC20-12-6	20	12	6	LAC30-12-21	30	12	21
LAC20-12-12	20	12	12	LAC30-12-24	30	12	24
LAC20-12-18	20	12	18	LAC30-12-27	30	12	27
LAC20-12-24	20	12	24	LAC30-12-30	30	12	30
LAC20-12-30	20	12	30	LAC30-12-33	30	12	33
LAC20-12-36	20	12	36	LAC40-1-0	40	1	0
LAC20-12-42	20	12	42	LAC40-1-3	40	1	3
LAC30-1-0	30	1	0	LAC40-1-6	40	1	6
LAC30-1-3	30	1	3	LAC40-1-9	40	1	9
LAC30-1-6	30	1	6	LAC40-1-12	40	1	12
LAC30-1-9	30	1	9	LAC40-1-15	40	1	15
LAC30-1-12	30	1	12	LAC40-12-0	40	12	0
LAC30-1-15	30	1	15	LAC40-12-3	40	12	3
LAC30-1-18	30	1	18	LAC40-12-6	40	12	6
LAC30-1-21	30	1	21	LAC40-12-9	40	12	9
LAC30-1-24	30	1	24	LAC40-12-12	40	12	12
LAC30-1-27	30	1	27	LAC40-12-15	40	12	15

**Table 4:** Mechanical properties of test specimens.

Specimen	*σ*_u_/Mpa	Specimen	*σ*_u_/Mpa	Specimen	*σ*_u_/Mpa
LAC20-1-00	18.10	LAC30-1-00	19.03	LAC30-12-18	93.88
LAC20-1-06	54.30	LAC30-1-03	44.43	LAC30-12-21	108.49
LAC20-1-12	75.92	LAC30-1-06	56.01	LAC30-12-24	115.76
LAC20-1-18	101.93	LAC30-1-09	64.34	LAC30-12-27	126.55
LAC20-1-24	115.61	LAC30-1-12	77.45	LAC30-12-30	136.52
LAC20-1-30	133.65	LAC30-1-15	87.76	LAC30-12-33	140.43
LAC20-1-36	151.90	LAC30-1-18	93.79	LAC40-1-00	29.27
LAC20-1-42	167.17	LAC30-1-21	100.36	LAC40-1-03	44.55
LAC20-12-00	18.09	LAC30-1-24	114.11	LAC40-1-06	58.26
LAC20-12-06	54.29	LAC30-1-27	115.75	LAC40-1-09	65.95
LAC20-12-12	73.64	LAC30-1-30	130.57	LAC40-1-12	76.98
LAC20-12-18	92.25	LAC30-1-33	138.43	LAC40-1-15	88.06
LAC20-12-24	109.49	LAC30-12-00	22.11	LAC40-12-00	28.07
LAC20-12-30	131.77	LAC30-12-03	46.83	LAC40-12-03	50.26
LAC20-12-36	145.72	LAC30-12-06	59.02	LAC40-12-06	61.84
LAC20-12-42	162.35	LAC30-12-09	71.25	LAC40-12-09	70.09
		LAC30-12-12	79.02	LAC40-12-12	76.73
		LAC30-12-15	88.91	LAC40-12-15	91.65

**Table 5:** Fitting coefficient of constitutive relation under triaxial compression.

Curve Branch	Confining Pressure Value	Fit Coefficient
Ascending Curve	0 MPa < *σ*_w_ ≤ 3 MPa	*α* = 1.712
	3 MPa < *σ*_w_ ≤ 9 MP	*α* = 2.043
	*σ*_w_ > 9 MPa	*α* = 3.120
descending branch	0 MPa < *σ*_w_ ≤ 3 MPa	*β* = 0.32 T + 0.548 (T = 1, 12)
	3 MPa < *σ*_w_ ≤ 9 MP	*β* = 0.474
	*σ*_w_ > 9 MPa	descending branch

Note: T represents the soaking time of aggregate (T = 1 h or 12 h).

## Data Availability

The data presented in this study are available on request from the corresponding author.

## Nomenclature

a, *μ*	Curve-fitting constants in Equation (2)
*φ*, *γ*	Curve-fitting constants in Equation (3)
*ζ*, *Φ*	Curve-fitting constants in Equation (7)
*k*, *q*	Curve-fitting constants in Equation (8)
*λ*, *ξ*	Curve-fitting constants in Equation (9)
*c*	Cohesion of lightweight aggregate concrete
*σ*_1_, *σ*_v_	Axial stress
*σ*_2_, *σ*_3_, *σ*_w_	Confining Pressure
*σ*_u_	Peak stress
*σ*_0_	Uniaxial compressive strength of concrete
*σ*	Stress
$\bar{\sigma }$	Octahedral normal stress
*τ*	Shear stress
*τ*_u_	Peak shear stress
*ε*	Axial strain
*ε*_u_	Uniaxial compression strain
LAC	Lightweight aggregate concrete
NAC	Natural aggregate concrete
LAC20	Design strength of 20 MPa lightweight aggregate concrete
LAC30	Design strength of 30 MPa lightweight aggregate concrete
LAC40	Design strength of 40 MPa lightweight aggregate concrete
